# Crosstalk between lactylation and RNA modifications in tumorigenesis: mechanisms and therapeutic implications

**DOI:** 10.1186/s40364-025-00824-9

**Published:** 2025-08-26

**Authors:** Shantong Liu, Qianquan Ma, Chong Zeng, Haoyu Li, Jun Su, Zhihao Song, Ruyu Yan, Zijin Zhao, Songhai Tian, Wei Huang

**Affiliations:** 1 Department of Neurosurgery, Xiangya Hospital, Central South University, Changsha, China; 2 Research Center of Carcinogenesis and Targeted Therapy, Xiangya Hospital, Central South University, Changsha, China; 3 National Clinical Research Center of Geriatric Disorders, Xiangya Hospital, Central South University, Changsha, China; 4 Department of Neurosurgery, Peking University Third Hospital, Peking University, Beijing, China; 5 Department of Respiratory and Critical Care Medicine, The Seventh Affiliated Hospital, Hengyang Medical School, University of South China, Changsha, China; 6 Clinical Research Center For Skull Base Surgery and Neurooncology In Hunan Province, Changsha, China; 7 Department of Neurosurgery, Hunan Children’s Hospital and the Affiliated Cancer Hospital of Xiangya School of Medicine, Central South University, Changsha, China; 8 State Key Laboratory of Natural and Biomimetic Drugs, Department of Molecular and Cellular Pharmacology, School of Pharmaceutical Sciences, Peking University, Beijing, China

**Keywords:** Lactylation modification, RNA modification, Crosstalk, Positive feedback loop, Targeted therapy

## Abstract

Complex crosstalk occurs between protein and nucleic acid modifications, with lactylation, an emerging post-translational modification (PTM), being implicated in tumor progression. However, the mechanisms mediating the crosstalk between lactylation and RNA modifications and their roles in disease pathogenesis remain largely unresolved. In this review, we summarize current advances in the regulatory interactions between lactylation and RNA modifications, explore their functional implications in cancer biology, and discuss the therapeutic potential of targeting these modifications individually or in combination. This work aims to provide a comprehensive overview of their mechanistic involvement in cancer and to inform novel strategies for precision-targeted therapy.

## Introduction

Epigenetic modifications drive tumorigenesis and cancer progression by orchestrating gene expression and chromatin dynamics [1, 2]. With advances in epigenetic research, nucleic acid modifications—including DNA and RNA methylation—along with emerging histone protein modifications have garnered significant interest. These modifications regulate transcription and translation while modulating cellular metabolism and are integral to both physiological homeostasis and disease pathogenesis [3]. Metabolically, protein and nucleic acid modifications converge on shared molecular substrates, establishing highly orchestrated interdependent networks [4, 5]. Recent studies have revealed intricate crosstalk between post-translational protein modifications (e.g., acetylation and ubiquitination) and RNA modifications (e.g., m6A and m5C), underscoring their pivotal roles in cancer, neurodegenerative, and cardiovascular diseases [6–8].

Unlike normal cells, tumor cells preferentially consume glucose to produce lactate via glycolysis, even under sufficient oxygen—aerobic glycolysis or the Warburg effect [9]. This metabolic reprogramming is closely linked to the malignant phenotype, meeting energy and biosynthetic demands while promoting cell survival, redox homeostasis, and adaptation to hypoxia [10–12]. In addition to its metabolic role, lactate acts as a signaling molecule, driving immune regulation, tumor microenvironment (TME) remodeling, and metabolic disease progression through pH-dependent and receptor-mediated mechanisms [13–15]. Lactate accumulation induces histone lactylation (e.g., H3K18la and H3K9la), which remodels chromatin and epigenetically regulates gene expression [16]. In addition to histones, lactylation also modifies effector proteins, altering their functions and interactions.

Lactylation, a recently identified post-translational modification, directly links lactate metabolism to gene regulation and has emerged as a key focus in cancer research. Moreover, RNA modifications are increasingly recognized for their roles in tumorigenesis and progression. The interplay between these modifications at the epigenetic and post-transcriptional levels may bridge tumor metabolic reprogramming and malignant phenotypes [17]. Lactylation modulates the expression, activity, binding, and stability of RNA-modifying proteins, whereas RNA modifications regulate lactylation by influencing glycolysis in tumors [18]. This review examines the mechanistic crosstalk between lactylation and RNA modifications in cancer, highlighting their integration in metabolic reprogramming and epigenetic regulation and offering new insights into tumor heterogeneity, immune evasion, and metabolic adaptation.

## Lactylation and RNA modifications

### Glycolysis and lactylation

Glycolysis converts glucose into pyruvate through a cascade of enzymatic reactions, generating ATP and NADH. The process begins with hexokinase (HK) phosphorylating glucose to glucose-6-phosphate. Phosphofructokinase-1 (PFK-1), the key rate-limiting enzyme, catalyzes the conversion of fructose-6-phosphate to fructose-1,6-bisphosphate (F1,6BP). PFK-1 exists in three isoforms: PFKP (platelet), PFKM (muscle), and PFKL (liver). Aldolase then cleaves F1,6BP into dihydroxyacetone phosphate and glyceraldehyde-3-phosphate. In the later stages, enolase converts 2-phosphoglycerate (2-PGA) to phosphoenolpyruvate (PEP), followed by pyruvate kinase (PK), which catalyzes pyruvate formation from PEP, coupled with ATP generation. Tumor cells predominantly express PKM2, a key regulator of glycolytic flux. Lactate dehydrogenase (LDH) catalyzes the reversible interconversion of pyruvate and lactate, with LDHA, the dominant isoform in tumors, promoting pyruvate-to-lactate conversion.

Lactate, a byproduct of glycolysis, serves as a crucial substrate for lysine lactylation, initiating lactylation. The hallmarks of lactylation are closely linked to metabolic remodeling in tumor cells and self-reinforcing positive-feedback mechanisms. Under physiological conditions, the serum lactate concentration ranges from 1.5 to 3 mM, whereas in cancer cells, it can increase to 10–30 mM, which is necessary to drive lactylation [19, 20]. Tumor cells sustain high lactate production through enhanced glycolytic flux, ensuring a continuous supply of substrates for lactylation [21]. For example, the cellular myelocytomatosis oncogene (c-Myc) and hypoxia-inducible factor 1α (HIF-1α) upregulate key glycolytic genes, including glucose transporter (GLUT), HK, glyceraldehyde-3-phosphate dehydrogenase (GAPDH), and LDH, thereby promoting glycolytic metabolism [22–24]. In parallel, the AKT oncogene enhances glycolysis by activating hexokinase 2 (HK2) and phosphofructokinases (PFKFB2, PFKFB3) and facilitating the plasma membrane localization of GLUT1 [25, 26]. Additionally, RAS activation induces GLUT1 expression, further increasing lactate levels [27].

Lactate accumulation continuously amplifies histone lactylation through a self-reinforcing positive feedback loop [28, 29]. Specifically, lactate promotes the transcription of glycolysis-related genes via histone lactylation, thereby increasing lactate production and establishing a metabolic‒epigenetic coupling loop of “glycolysis‒lactate production‒lactylation”. In pancreatic ductal adenocarcinoma (PDAC), H3K18 lactylation (H3K18la) transcriptionally upregulates TTK protein kinase (*TTK*) and BUB1 mitotic checkpoint serine/threonine kinase B (*BUB1B*), with TTK enhancing LDHA activity, thereby promoting glycolysis, increasing lactate levels, and reinforcing histone lactylation. This forms a tumor-promoting feedback loop of lactylation modifications in PDAC [30]. Similarly, in clear cell renal cell carcinoma (ccRCC), histone lactylation is enriched in the *PDGFRβ* promoter region and activates its transcription, upregulating LDHA and GLUT1, which increases lactate production and histone lactylation [28]. In hepatocellular carcinoma (HCC), lactate accumulation induces H3K18la, which further upregulates SRSF10, indirectly increasing GLUT1, HK1, and LDHA expression, thereby sustaining lactate accumulation and histone lactylation [29]. Another positive feedback mechanism operates at the regulatory nodes of lactylation modification and involves lactyltransferases. Alanyl-tRNA synthetase 1 (AARS1) catalyzes lactylation at the YAP K90 site and TEAD1 K108 site, activating the YAP/TEAD transcriptional complex. In turn, YAP–TEAD1 transactivates the transcription of *AARS1*, forming a self-sustaining loop [31]. Targeting these key regulatory nodes may provide a strategy to disrupt the vicious cycle of tumor metabolism.

### Lactylation modification

In 2019, Prof. Yingming Zhao’s team first reported histone lysine L-lactylation (KL-la), identifying 28 lactylation sites in the core histones of human and mouse cells [32]. Hai-Ping Hao and Hui Ye’s team subsequently developed the cyclic immonium ion tracer technique to detect protein lactylation, further confirming lysine lactylation modification [33, 34]. In addition to histones, lactylation occurs on transcription factors, enzymes, and mitochondrial proteins, directly regulating protein conformation and function [35, 36].

Lactylation occurs via both enzymatic and non-enzymatic mechanisms involving the covalent modification of lysine residues by lactate through intermediates such as S-D-lactoylglutathione (LGSH/SLG) or lactyl-coenzyme A (Lactyl-CoA). Enzymatically, lactyltransferases catalyze lysine L-lactylation by transferring lactoyl groups from lactyl-CoA to protein lysine residues. Lysine L-lactylation is the predominant lactylation form induced by glycolysis and plays a crucial role in metabolic‒epigenetic regulation [37]. The identified lactyltransferases include p300/CBP [38], GCN5 [39], KAT8 [40], ESCO1/2 [41, 42] and ATAT1 [43]. Notably, alanyl-tRNA synthetases 1 and 2 (AARS1/2) can directly utilize lactate as a substrate to mediate the lactylation of diverse proteins [44], including p53 [45], YAP [31], cGAS [46] and mitochondrial proteins [47]. Conversely, deacetylases such as HDAC1-3 and SIRT1-3 remove lactoyl groups from target proteins [48]. Non-enzymatic lactylation occurs via nucleophilic substitution (SN reaction) between LGSH and lysine residues, leading to lysine D-lactylation (KD-la). LGSH, a byproduct of the glyoxalase pathway, accumulates under conditions of enhanced glycolysis or glyoxalase system imbalance, driving lactylation. This modification plays a key role in modulating inflammatory responses and maintaining immune homeostasis [49, 50].

Lactylation modifications are characterized by a dual mechanism regulating gene transcription and protein function. At the transcriptional level, histone lactylation activates gene expression through selective enrichment in the promoter regions of target genes. In glioblastoma (GBM), p300 catalyzes lactate-driven histone lactylation in the promoter region of interleukin 10 (*IL-10*), increasing IL-10 expression, which inhibits T-cell activity and facilitates tumor immune escape [51]. At the protein level, non-histone lactylation dynamically regulates protein localization, binding capacity, stability, and enzymatic activity. AARS1, which acts as a lactate sensor, binds lactate to form lactate-AMP, which is then transferred to the K120/K139 sites of p53. Lactylation of the DNA-binding domain of p53 impairs its liquid‒liquid phase separation (LLPS), DNA binding, and transcriptional activation, thereby promoting tumorigenesis [45].

In tumors, lactylation modifications regulate malignant phenotypes, including cell proliferation, immune evasion, and remodeling of the immunosuppressive microenvironment (ISME), by modifying key effector proteins. In acute myeloid leukemia (AML), lactate accumulation induces H4K5 lactylation at the *PD-L1* promoter, inducing *PD-L1* transcription, thereby inhibiting CD8 + T-cell activation and establishing an immunosuppressive microenvironment [52]. Additionally, lactylation contributes to chemoresistance by enhancing DNA repair. In gastric cancer (GC), lactylation at the K388 site of NBS1 promotes homologous recombination (HR) by facilitating the formation of the MRE11-RAD50-NBS1 (MRN) complex and DNA repair protein aggregation at the damage site, inducing chemoresistance [53].

### RNA modification

**Fig. 1:**
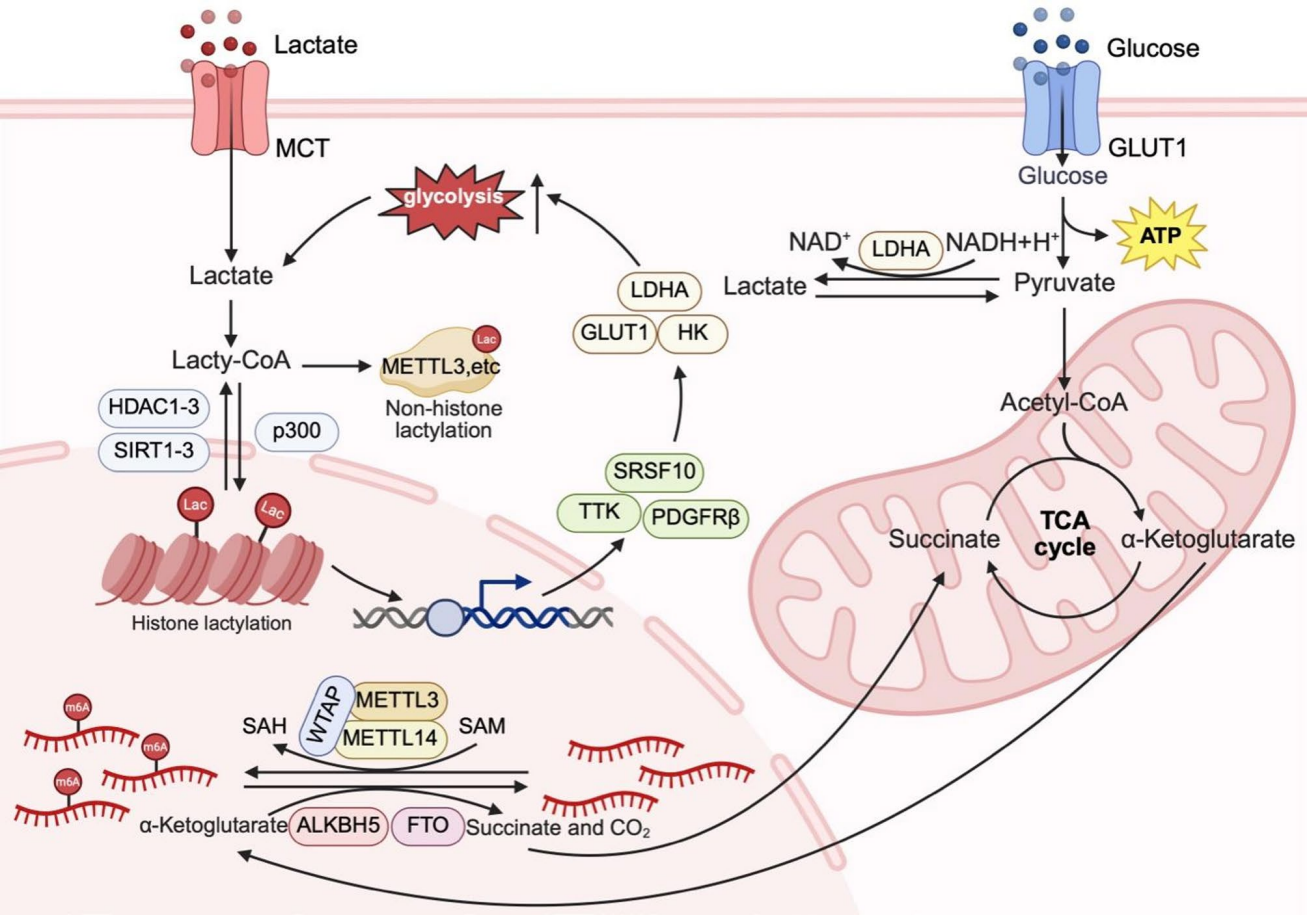
Lactylation and RNA modification at the metabolic level. Tumor-associated metabolic reprogramming promotes aerobic glycolysis and increases lactate production. Lactate is converted into lactyl-CoA, which serves as a substrate for both histone and non-histone lactylation mediated by writers such as p300. Histone lactylation enhances glycolysis and lactate production by promoting the transcription of glycolytic genes, thereby establishing a self-reinforcing positive feedback loop. In parallel, N^6^-methyladenosine (m6A) is dynamically regulated by the activities of methyltransferases (e.g., METTL3 and METTL14) and demethylases (e.g., FTO and ALKBH5), whose functions are dependent on metabolic intermediates. S-Adenosylmethionine (SAM) donates methyl groups for RNA methylation, whereas α-ketoglutarate (α-KG), a product of the tricarboxylic acid (TCA) cycle, acts as a cofactor for RNA demethylation. Created in BioRender. Liu, S. (2025) https://BioRender.com/a6b218d

RNA modifications, including N6-methyladenosine (m6A), 5-methylcytidine (m5C), N4-acetylcytosine (ac4C), and N1-methyladenosine (m1A), are covalent chemical alterations that regulate RNA structure and function. Notably, RNA glycosylation has recently emerged as a novel RNA modification, predominantly localized on the cell membrane and involved in signal transduction, and its discovery by Nobel laureate Carolyn Bertozzi underscores its potential biological significance [54–56]. Among these, m6A modifications are dynamically regulated by writer enzymes (e.g., the methyltransferase complex comprising METTL3/METTL14/WTAP), eraser enzymes (e.g., FTO and ALKBH5), and reader proteins (e.g., the YTHDF family) [57]. By modulating RNA stability, translation, subcellular localization, and splicing, RNA modifications drive key oncogenic processes, including metabolic reprogramming, proliferation, invasion, immune evasion, and drug resistance [58]. For example, RNA methyltransferase METTL3-mediated m6A modification enhances the translation of the oncogenes *EGFR* and *TAZ*, thereby promoting tumor cell proliferation, survival, and invasion [59]. Both lactylation and RNA modifications are regulated by tumor metabolic reprogramming. The activities of RNA-modifying enzymes (e.g., FTO and ALKBH5) are modulated by S-adenosylmethionine (SAM) and α-ketoglutarate (α-KG): SAM donates methyl groups for methylation [60], whereas α-KG serves as a demethylase cofactor, regulating RNA modification dynamics [61]. Shifts in the α-KG/SAM ratio induced by tumor metabolic reprogramming reshape RNA modifications. Simultaneously, hyperglycolysis-derived lactate fuels lactylation, reinforcing epigenetic remodeling (Fig. 1). This interplay, with metabolic reprogramming as a central hub, highlights a critical layer of tumor progression. However, the regulatory crosstalk between RNA and lactylation modifications at the metabolic level remains unclear, necessitating experimental investigation and multidimensional analyses.

## Mechanisms of crosstalk between lactylation and RNA modifications

Emerging experimental evidence reveals the crosstalk network between lactylation modification and RNA modification, which is orchestrated through two mutually exclusive pathways: Epigenetic cascade regulation—one modification can achieve trans-regulation of the expression of enzymes involved in the other modification by altering the epigenetic state of their encoding genes (e.g., histone lactylation influences the chromatin accessibility of genes encoding m6A methyltransferases); covalent modification of enzyme function—through post-translational modification of specific structural domains (e.g., lactylation modification of RNA methyltransferases), the conformational stability, catalytic activity, and substrate-binding capacity of enzyme proteins are directly affected, forming a dynamic regulatory axis between post-translational modifications and enzyme function [44]. This intricate, multilayered regulatory network provides a framework for advancing epigenetics-based precision regulation.

### Transcriptional regulation: histone lactylation in RNA modifications

Histone lactylation, particularly H3K18la, remodels chromatin by enriching in the promoters of target genes, thereby activating the transcription of key RNA modification regulators, including writers, readers and erasers, and orchestrating RNA modification-dependent control of mRNA stability and translation. Specifically, H3K18la promotes RNA methyltransferase transcription, thereby enhancing RNA modifications. In sepsis-associated lung injury, p300-mediated H3K18la significantly upregulates METTL3 expression via enrichment in the promoter region of *METTL3*, driving ferroptosis in lung epithelial cells via m6A-dependent stabilization of *ACSL4* mRNA [62]. A similar mechanism applies to METTL14, where H3K18la facilitates its transcription through epigenetic remodeling. METTL14-mediated m6A modification of *ATF5* mRNA subsequently accelerates its degradation in an m6A-dependent manner [63]. Similarly, in colorectal cancer (CRC), NSUN2, an m5C methyltransferase, is transcriptionally activated by H3K18la [64].

In addition to modulating RNA methyltransferases, histone lactylation also regulates RNA modification reader proteins. In PDAC, p300-mediated H3K18la enrichment at the *IGF2BP2* promoter enhances its transcription, stabilizing *CSF1* and *MYC* mRNAs in an m6A-dependent manner to drive tumor progression [65]. The YTH structural domain proteins are also modulated by lactylation modifications. In HCC, lactate accumulation induces *YTHDC1* transcription through H3K18la, leading to increased expression. Upregulated YTHDC1 stabilizes the lncRNA NEAT1, which activates stearoyl-CoA desaturase (SCD), disrupting lipid metabolism and promoting tumorigenesis [66]. Similarly, p300-mediated H3K18la upregulates YTHDF2, modulating RNA degradation via both m6A-dependent and m6A-independent mechanisms [17, 67]. In lung fibrosis, H3K18la enhances the transcription of *YTHDF1*, which binds to m6A-modified *Nrep* mRNA, stabilizing it and increasing its translation efficiency, thereby accelerating fibroblast-to-myofibroblast transformation (FMT) [68].

Histone lactylation also modulates RNA demethylases, ensuring the dynamic regulation of RNA modifications. In G6PT deficiency, lactate accumulation upregulates ALKBH5 expression through p300-mediated H3K18la. ALKBH5 decreases the stability of *NLRP3* mRNA by removing its m6A modification, thereby inhibiting macrophage NLRP3 inflammasome activation and weakening host resistance to infection [69]. Similarly, H3K18la enhances *ALKBH3* transcription via enrichment at the *ALKBH3* promoter [70]. Retinopathy is a common complication of diabetes, and emerging evidence suggests that p300-mediated H3K18la increases FTO expression, reduces the m6A modification of *CDK2* mRNA, and enhances its stability via the m6A-YTHDF2 axis, thereby driving pathological retinal angiogenesis [71].

Collectively, these findings highlight lactate not only as a metabolic byproduct but also as a pivotal signaling molecule that regulates the transcription of RNA modification enzymes through histone lactylation. This intricate metabolic‒epigenetic regulatory network provides new insights into disease pathogenesis and therapeutic strategies.

### Post-translational regulation: lactylation of RNA modifications

Lactate accumulation in the TME or under metabolic stress has been shown to directly regulate the function and stability of RNA-modifying enzymes via lactylation, thereby modulating the dynamic regulation of RNA modifications. Lactylation enhances the RNA-binding capacity of methyltransferases. In the TME, lactate-induced lactylation of METTL3 at K281 and K345 within its zinc-finger domain (ZFD) strengthens its affinity for m6A-modified RNAs, promoting m6A-mediated immunosuppression and ultimately facilitating tumor immune evasion [72]. Similarly, tumor-derived lactate induces NSUN2 lactylation at K356, increasing its binding ability to specific m5C-modified RNAs and driving m5C-dependent metabolic reprogramming to support CRC invasion and metastasis [64]. In addition to enhancing RNA binding, lactylation also regulates methyltransferase activity by inducing conformational changes. In GC cells, elevated copper concentrations trigger lactylation of METTL16 at K229, activating its methyltransferase activity by weakening the autoinhibited state. Activated METTL16 catalyzes m6A modification on *FDX1* mRNA, stabilizing its transcript and promoting tumor cell cuproptosis, whereas SIRT2-mediated delactylation reverses this process [73]. A similar regulatory mechanism has been observed in the ac4C modification system. During Kaposi’s sarcoma-associated herpesvirus (KSHV) infection, α-tubulin acetyltransferase 1 (ATAT1) catalyzes the lactylation of NAT10 at K290, increasing its RNA acetyltransferase activity and facilitating its interaction with THUMPD1. This interaction elevates tRNA^Ser−CGA−1−1^ ac4C modification levels to increase the translation efficiency of viral mRNAs, thereby enhancing viral lysis and replication [43].

By recognizing and binding to specific RNA modification sites, RNA modification reader proteins regulate gene expression. Emerging evidence indicates that lactylation dynamically modulates the stability and RNA-binding capacity of RNA modification reader proteins. During severe fever with thrombocytopenia syndrome virus (SFTSV) infection, establishment of sister chromatid cohesion N-acetyltransferase 1 (ESCO1) functions as an acetyltransferase, catalyzing the lactylation of the m6A reader protein YTHDF1 at K517 and K521. This modification promotes YTHDF1 ubiquitylation, leading to a significant reduction in its protein stability. As a result, the diminished stability of YTHDF1 weakens the degradation of m6A-modified viral mRNAs, thereby enhancing the stability and translational efficiency of viral mRNAs and ultimately promoting viral replication [41]. Additionally, hyperglycolysis-induced lactate accumulation drives the lysine lactylation of IGF2BP3 at the K76 site (IGF2BP3lac). This modification enhances the binding of IGF2BP3 to m6A-modified *PCK2* and *NRF2* mRNAs, thereby significantly increasing their expression and promoting lenvatinib resistance in HCC [74].

RNA-modifying erasers dynamically regulate RNA stability and translational efficiency through reversible demethylation, and their function is directly modulated by lactylation. During infection with DNA viruses, including herpes simplex virus (HSV-1) and monkeypox virus (MPXV), lactylation at K284 of ALKBH5 significantly enhances its binding affinity to interferon-beta (*IFN-β*) mRNA, thereby promoting the demethylation of its m6A modifications. This process increases *IFN-β* mRNA biogenesis, ultimately strengthening the host’s innate immune response. Moreover, the acetyltransferase ESCO2 and deacetylase SIRT6 serve as the “writer” and “eraser” of the ALKBH5 lactylation modification, respectively, fine-tuning its lactylation modification status [42] (Table 1). Protein lactylation modulates the function of RNA modification enzymes, bridging metabolic activity and post-transcriptional regulation at the protein level.

Notably, lactylation of the Alternative Polyadenylation (APA) regulator NUDT21 at K23 enhances its interaction with CPSF6 and promotes CFIm complex assembly, leading to 3’ UTR lengthening of FDX1 transcripts and reduced protein output, thereby promoting cuproptosis resistance in esophageal squamous cell carcinoma (ESCC) cells [75].

**Table 1:** Mechanisms of lactylation regulating RNA-modifying proteins and their biological implications

Regulation Mechanism	Regulator (Type)	RNA modifying Protein (Type)	Target Site	Genomic Location	Mechanisms of lactylation regulation	Biological Implications	Refs.
**Transcriptional Regulation**	EP300 (*Writer*)	YTHDF2 (*Reader*)	\	YTHDF2 promoter region	H3K18la promotes YTHDF2 expression	Induces degradation of PER1 and TP53 mRNAs (m6A), promoting OM tumorigenesis	[17]
	p300 (*Writer*)	NSUN2 (*Writer*)	\	NSUN2 promoter region	H3K18la activates NSUN2 transcription	Stabilizes ENO1 mRNA (m5C), promoting CRC progression	[64]
	EP 300 (*Writer*)	IGF2BP2 (*Reader*)	\	IGF2BP2 promoter region	H3K18la promotes IGF2BP2 transcription	Stabilizes CSF1 and MYC mRNAs (m6A), promoting PDAC progression	[65]
	\	YTHDC1 (*Reader*)	\	YTHDC1 promoter region	H3K18la promotes YTHDC1 expression	Stabilizes m6A-modified NEAT1, promoting HCC progression	[66]
	\	ALKBH3 (*Eraser*)	\	ALKBH3 promoter region	H3K18la promotes ALKBH3 expression	Reduces SP100A mRNA stability (m1A), promoting OM progression	[70]
	\	METTL14 (*Writer*)	\	METTL14 promoter region	H3K18la upregulates METTL14 expression	Promotes ATF5 mRNA degradation (m6A), inhibiting stemness in GC	[63]
	p300 (*Writer*)	METTL3 (*Writer*)	\	METTL3 promoter region	H3K18la promotes METTL3 expression	Enhances JAK1 m6A, promoting tumor immunosuppression in TIMs	[72]
	p300 (*Writer*)	ALKBH5 (*Eraser*)	\	\	H3K18la upregulates ALKBH5 expression	Attenuates NLRP3 mRNA stability (m6A), weakening host infection resistance	[69]
	p300/CBP (*Writer*)	METTL3 (*Writer*)	\	METTL3 promoter region	H3K18la promotes METTL3 expression	Stabilizes ACSL4 mRNA (m6A), promoting ferroptosis in sepsis-associated ALI	[62]
	\	YTHDF1 (*Reader*)	\	YTHDF1 promoter region	H3K18la activates YTHDF1 transcription	Stabilizes NREP protein (m6A), promoting As-IPF progression	[68]
	EP300 (*Writer*)	YTHDF2 (*Reader*)	\	YTHDF2 promoter region (B-site and C-site)	H3K18la promotes YTHDF2 expression	Upregulates G3BP1, promoting myocardial ischemia–reperfusion injury	[67]
	p300 (*Writer*)	FTO (*Eraser*)	\	FTO promoter region	H3K18la promotes FTO expression	Stabilizes CDK2 mRNA via m6A-YTHDF2 axis, promoting retinal angiogenesis	[71]
**Post-translational Regulation**	\	NSUN2 (*Writer*)	K356	\	Enhanced capture of target mRNA	Stabilizes ENO1 mRNA (m5C), promoting CRC progression	[64]
	AARS1 and AARS2 (*Writer*), SIRT2 (*Eraser*)	METTL16 (*Writer*)	K229	\	Enhanced methyltransferase activity	Increases FDX1 expression (m6A), promoting GC cell cuproptosis	[73]
	NAA10 (*Writer*)	NSUN2 (*Writer*)	K508	\	Enhanced methyltransferase activity	Stabilizes GCLC mRNA (m5C), promoting ferroptosis resistance	[76]
	\	METTL3 (*Writer*)	K281, K345	\	Enhanced capture of target mRNA	Promotes JAK1 m6A modification, enhancing immunosuppression in TIMs	[72]
	ESCO2 (*Writer*), SIRT6 (*Eraser*)	ALKBH5 (*Eraser*)	K284	\	Promoting binding to IFN-β mRNA	Increases IFN-β mRNA biogenesis (m6A), enhancing innate immune response	[42]
	\	IGF2BP3 (*Reader*)	K76	\	Enhanced binding to PCK2 and NRF2 mRNAs	Increases PCK2 and NRF2 expression (m6A), promoting lenvatinib resistance in HCC	[74]
	ATAT1 (*Writer*)	NAT10 (*Writer*)	K290	\	Enhanced acetyltransferase activity and the interaction of NAT10 with THUMPD1	Mediates tRNA^Ser−CGA−1−1^ ac4C modification, enhancing viral mRNA translation and KSHV reactivation	[43]
	ESCO1 (*Writer*), SIRT6 (*Eraser*)	YTHDF1 (*Reader*)	K517, K521	\	Promoting the degradation of YTHDF1	Weakens degradation of viral mRNAs (m6A), promoting viral replication	[41]
	AARS1 (*Writer*), HDAC2 (*Eraser*)	NUDT21 (*APA regulator*)	K23	\	Enhanced the interaction of NUDT21 with CPSF6	Destabilizes FDX1 mRNA, promoting cuproptosis resistance	[75]
**Metabolic Feedback**	p300 (*Writer*)	NSUN2 (*Writer*), YBX1 (*Reader*)	K356	NSUN2 promoter region	H3K18la activates NSUN2 transcription, enhanced capture of target mRNA	Stabilizes ENO1 mRNA (m5C), promoting lactate production and H3K18la	[64]
	\	METTL1 (*Writer*)	\	METTL1 promoter region	H3K9la activates METTL1 transcription	Upregulates PKM2 expression (m7G), promoting lactate production and H3K9la	[77]

### Epigenetic regulation: RNA modifications in lactylation

#### RNA modifications in lactate metabolism

As a substrate for lactylation, lactate induces this modification [13, 32]. Notably, intracellular lactate levels positively correlate with pan-lysine lactylation (Pan-Kla) levels in tumor cells [74, 77]. RNA modifications regulate lactate biosynthesis by modulating the mRNA stability and translational efficiency of key glycolytic enzymes, including HK, PFK-1, ALDOA, ENO1, and PK. In cervical cancer cells, METTL3 mediates m6A modification of the 3’-untranslated region (3’ UTR) of *HK2* mRNA, increasing its stability by recruiting YTHDF1, thereby driving the Warburg effect and promoting tumorigenesis [78]. Within the hypoxic tumor microenvironment, HIF-1α directly binds to the hypoxia-responsive element (HRE) of the *ALDOA* promoter, activating its transcription. Concurrently, FTO-mediated m6A demethylation reduces YTHDF2-dependent mRNA degradation, synergistically upregulates ALDOA expression, enhances glycolytic activity and lactate production, and ultimately facilitates HCC cell growth and migration [79]. In bladder cancer, the m5C modification reader ALYREF binds to m5C-modified sites within the 3’ UTR of *PKM2* mRNA, stabilizing its transcript and enhancing its expression. This modification increases glycolytic flux and promotes lactate accumulation [80].

Moreover, RNA modifications directly regulate LDH expression, thereby promoting lactate production. In CRC, METTL3-mediated m6A modification within the coding sequence (CDS) of *LDHA* mRNA enhances transcript stability and translation through YTHDF1 recruitment, significantly promoting glycolysis and lactate production [81]. Additionally, as an m6A reader, leucine-rich pentatricopeptide repeat containing protein (LRPPRC) binds to m6A-modified *LDHA* mRNA, further stabilizing the transcript and increasing its expression, ultimately promoting glycolysis and lactate production [82]. Moreover, RNA modifications regulate LDH expression indirectly through noncoding RNAs. Under glucose deprivation conditions, YTHDF3 promotes degradation of the long noncoding RNA (lncRNA) DICER1-AS1, alleviating its repression of glycolytic genes such as LDHA and HK2, thereby promoting glycolysis and lactate accumulation in pancreatic cancer cells [83]. In dendritic cells (DCs), YTHDF2 accelerates lnc-Dpf3 degradation, weakening its inhibitory impact on the HIF-1α/LDHA transcriptional axis and leading to increased glycolysis, lactate production, and cell migration [84].

In addition, RNA modifications regulate lactate levels by modulating the expression of GLUT1 and the monocarboxylate transporter (MCT) family. Elevated GLUT1 expression significantly enhances glucose uptake and glycolytic activity in tumor cells [85]. In CRC, METTL3 catalyzes m6A modification in both the 3’ UTR of GLUT1 and the 5’/3’ UTR of HK2, stabilizing their mRNAs via an IGF2BP2/3-dependent mechanism and thereby promoting glycolysis and lactate production [86]. Similarly, in HCC, FTO removes m6A modification from *GLUT1* mRNA, counteracting YTHDF2-mediated degradation, increasing mRNA stability and expression, and ultimately enhancing glycolysis and lactate production [87]. While MCTs are not directly involved in lactate synthesis, they play a pivotal role in modulating intracellular and extracellular lactate concentrations by facilitating their transmembrane transport. For example, in melanoma and CRC models, ALKBH5 enhances the stability of *MCT4* mRNA by removing its m6A modification, thereby promoting lactate efflux and increasing lactate accumulation in the TME [88].

As RNA modifications govern both lactate production and transport, they likely act as upstream regulators of lactylation. Unraveling this regulatory axis holds significant potential for future research.

#### RNA modification in lactylation

Despite growing interest in RNA modifications, their direct role in regulating protein lactylation remains largely unexplored. However, accumulating evidence suggests that RNA modifications can indirectly influence lactylation by modulating glycolytic metabolism. For example, in liver fibrosis, elevated IGF2BP2 expression enhances the stability and translational efficiency of *ALDOA* mRNA by recognizing its m6A modification site. This process significantly boosts glycolytic activity in hepatic stellate cells (HSCs), leading to lactate accumulation and ultimately H3K18 lactylation [89]. Additionally, the m4C methyltransferase METTL15 facilitates mitoribosome assembly and stability by catalyzing the m4C839 modification of 12 S rRNA. Loss of METTL15 impairs mitochondrial oxidative phosphorylation (OXPHOS), shifting cellular metabolism toward glycolysis and promoting excessive lactate secretion, which in turn induces H4K12 and H3K9 lactylation [90]. In CRC, the methyltransferase METTL1 promotes PKM2 expression and enhances glycolysis and lactate production by modifying *PKM* mRNA with N7-methylguanosine (m7G), thereby promoting Pan-Kla and H3K9la [77] (Table 2).

**Table 2:** RNA modification regulates lactate or lactylation

Classification	Regulator (Type)	Modification	Target (Site)	Mechanism	Outcome	Refs.
**Glycolytic Enzymes**						
**HK**	METTL3 (*Writer)*, IGF2BP2/3 (*Reader*)	m6A	HK2 and GLUT1(5’/3’UTR)	Enhancing HK2 and GLUT1 mRNAs stability	Enhancing glycolysis, lactate production and tumor progression	[86, 91–93]
	METTL3 (*Writer*), YTHDF1 (*Reader*)	m6A	HK2 (3’UTR)	Increasing HK2 mRNA stability	Promoting glycolysis, lactate production and CC cell proliferation	[78]
	KIAA1429 (*Writer*), HuR (*Reader*)	m6A	HK1 (CDS)	Jointly improving HK1 mRNA stability	Enhancing glycolysis and increasing sorafenib resistance in HCC	[94]
	RBM15 (*Writer*)	m6A	HK2 (3’ UTR)	Enhancing HK2, GPI, and PGK1 mRNAs stability	Promoting glycolysis and osteosarcoma progression	[95]
	IGF2BP2 (*Reader*), FTO and ALKBH5 (*Eraser*)	m6A	HK2	Increasing HK2 mRNA stability	Enhancing glycolysis and promoting tumor progression	[86, 96, 97]
**PFK-1**	YTHDC1 (*Reader*)	m6A	PFKM (CDS)	Enhancing the stability and translation of PFKM and LDHA mRNAs	Enhancing glycolysis, lactate production and promoting OS progression	[98]
	YTHDF2 (*Reader*), FTO (*Eraser*)	m6A	PFKP and LDHB	Enhancing PFKP and LDHB mRNAs stability	Enhancing glycolysis and promoting leukemia progression	[99]
**Aldolase**	YTHDF2 (*Reader*), FTO (*Eraser*)	m6A	ALDOA (CDS)	Enhancing ALDOA mRNA stability	Enhancing glycolysis and promoting HCC growth under hypoxia	[79]
	IGF2BP2 (*Reader*)	m6A	ALDOA	Enhancing ALDOA mRNA stability and translation	Enhancing glycolysis and histone lactylation, and promoting liver fibrosis	[89]
**Enolase**	METTL3 (*Writer*), YTHDF1 (*Reader*), ALKBH5 (*Eraser*)	m6A	ENO1 (ORF 359 A)	Enhancing ENO1 mRNA translation	Promoting glycolysis, lactate production and LUAD tumorigenesis	[100]
	KIAA1429 (*Writer*)	m6A	ENO1 (CDS 2273–2278)	Enhancing ENO1 mRNA stability	Promoting aerobic glycolysis and OC progression	[101]
	WTAP (*Writer*)	m6A	ENO1 (3’UTR)	Promoting ENO1 mRNA stability	Promoting breast cancer glycolysis	[102]
	METTL3 (*Writer*)	m6A	LINC00520 (183nt, 710nt)	Indirectly promoting ENO1 stability	Enhancing glycolysis, lactate production and inducing cisplatin resistance in OS	[103]
**PK**	HNRNPC (*Reader*)	m6A	PKM (motif GGACU near exon 9)	Regulating PKM alternative splicing and promoting PKM2 expression	Promoting glycolysis, lactate production and progression in PTC	[104]
	ALYREF (*Reader*)	m5C	PKM2 (3’UTR)	Enhancing PKM2 mRNA stability	Promoting glycolysis, lactate production and bladder cancer cell proliferation	[80]
	METTL1 (*Writer*)	m7G	PKM	Increasing PKM mRNA stability	Enhancing glycolysis and promoting immune evasion in CRC	[77]
	METTL14 (*Writer*), IGF2BP (*Reader*), ALKBH5 (*Eraser*)	m6A	JMJD8 (3’UTR)	Enhancing the enzymatic activity of PKM2	Enhancing glycolysis, lactate production and promoting CRC progression	[105]
**PDK4**	IGF2BP3 (*Reader*), YTHDF1 (*Reader*)	m6A	PDK4 (5’UTR)	Enhancing PDK4 mRNA stability and translation	Enhancing glycolysis, lactate production	[106]
	METTL16 (*Writer*), IGF2BP1 (*Reader*)	m6A	SOGA1	Indirectly upregulating PDK4 expression	Promoting glycolysis and CRC progression	[107]
**LDH**	IGF2BP3 (*Reader*)	m6A	LDHA (3’UTR)	Enhancing LDHA mRNA stability	Boosting lactate production and GC immune evasion	[108]
	LRPPRC (*Reader*)	m6A	LDHA (3’UTR)	Enhancing LDHA mRNA stability	Enhancing glycolysis and TNBC progression	[82]
	METTL3 (*Writer*), YTHDF1 (*Reader*)	m6A	LDHA (CDS)	Promoting LDHA mRNA translation	Promoting glycolysis, tumor progression and 5-FU resistance	[81, 109, 110]
	NAT10 (*Writer*)	ac4C	JunB	Indirectly upregulating LDHA expression	Enhancing glycolysis and promoting TNBC progression and immunosuppression	[111]
	YTHDF2 (*Reader*)	m6A	Inc-Dpf3 (3812–3815 nt and 3852–3855 nt)	Indirectly upregulating LDHA expression	Promoting dendritic cell glycolysis metabolism and migration	[84]
	YTHDF3 (*Reader*)	m6A	lncRNA DICER1-AS1 (exon region)	Indirectly promoting LDHA, HK2 expression	Promoting glycolysis, lactate production and pancreatic cancer progression	[83]
**Transporters**						
**GLUT1**	METTL3 (*Writer*)	m6A	GLUT1 (CDS/3 ‘UTR)	Enhancing GLUT1 mRNA translation	Enhancing glycolysis, lactate production and promoting tumor progression	[112, 113]
	METTL3 (*Writer*)	m6A	GLUT1	Enhancing GLUT1 mRNA stability	Promoting lactate production, progression and resistance to mTOR inhibitors in ccRCC	[114]
	WTAP (*Writer*), IGF2BP2 (*Reader*)	m6A	PIGT (3 ‘UTR)	Promoting GLUT1 glycosylation and membrane trafficking	Promoting glycolysis, proliferation and metastasis of bladder cancer cells	[115]
	YTHDF2 (*Reader*), FTO (*Eraser*)	m6A	GLUT1 and PKM2	Enhancing GLUT1, PKM2 and c-Myc mRNAs stability	Promoting glycolysis and progression in HCC	[87]
**MCT**	ALKBH5 (*Eraser*)	m6A	MCT4	Enhancing the MCT4 mRNA stability	Promoting lactate production and the infiltration of Tregs and MDSCs in the TME	[88]

### Positive feedback loops: lactylation and RNA modification

**Fig. 2:**
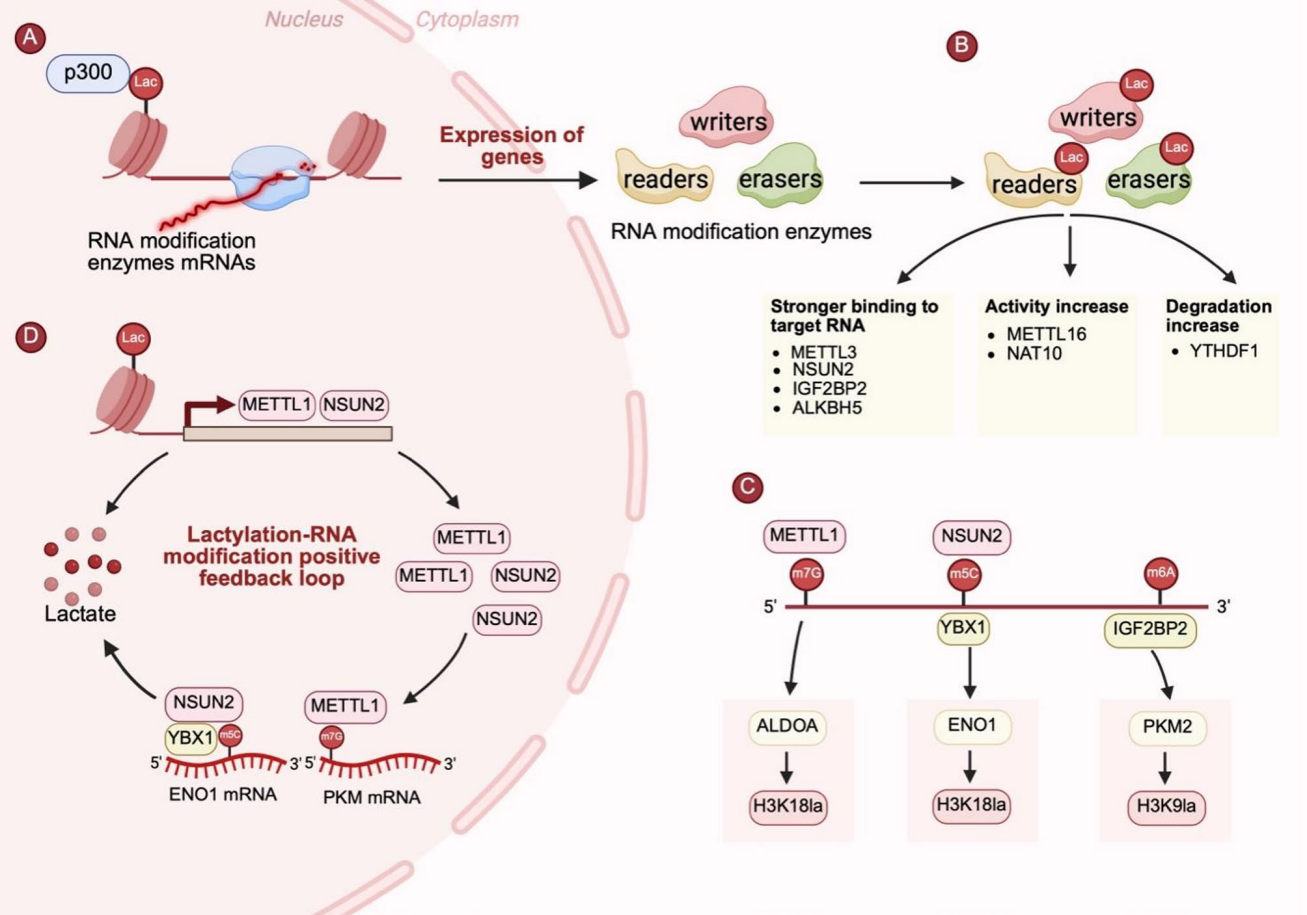
Crosstalk between lactylation and RNA modifications. **(A)** Histone lactylation promotes the transcription of RNA modification enzymes by enriching the promoters of target genes. **(B)** Lactylation of RNA modification enzymes, including writers, readers, and erasers, modulates their function by altering RNA-binding affinity (e.g., METTL3, NSUN2, IGF2BP2, ALKBH5), catalytic activity (e.g., METTL16, NAT10), or protein stability (e.g., YTHDF1). **(C)** RNA modifications regulated by METTL1 (m7G), NSUN2 (m5C), and IGF2BP2 (m6A) increase glycolytic gene expression, thereby promoting lactate production and subsequently driving histone lactylation. **(D)** A positive feedback loop is established wherein histone lactylation induces the transcription of RNA modification enzymes, which in turn promote glycolytic gene expression and lactate production via RNA modification, thereby reinforcing both lactylation and RNA modification. Created in BioRender. Liu, S. (2025) https://BioRender.com/s2voqxr

Recent studies have revealed a positive feedback loop between lactylation and RNA modifications, which synergistically drive tumor metabolic reprogramming and progression. For example, NSUN2 is highly expressed in colorectal cancer (CRC), where it catalyzes m5C modification of *ENO1* mRNA and enhances its stability via the reader protein YBX1, thereby promoting glycolysis and lactate production. Notably, lactate accumulation subsequently induces H3K18 lactylation, which in turn activates *NSUN2* transcription, forming an NSUN2/YBX1/m5C-ENO1 feedback loop that sustains CRC progression [64]. Similarly, in CRC, METTL1 catalyzes the m7G modification of *PKM* mRNA, increasing PKM2 expression and enhancing aerobic glycolysis and lactate synthesis. The resulting lactate accumulation transactivates METTL1 expression via H3K9 lactylation, establishing a METTL1-PKM2-H3K9la metabolic‒epigenetic regulatory loop that promotes tumor proliferation [77]. These findings underscore the intricate crosstalk between metabolism and epigenetic regulation, providing a theoretical foundation for the development of multidimensional antitumor strategies targeting the lactylation‒RNA modification axis (Fig. 2).

## Lactylation-RNA modification in tumors

### Tumorigenesis and progression

Lactylation–RNA modification crosstalk contributes to tumorigenesis by repressing tumor suppressor gene expression. For example, in melanoma, H3K18la upregulates YTHDF2, which facilitates the degradation of m6A-modified *PER1* and *TP53* mRNAs by recognizing their m6A modification sites. This degradation attenuates the tumor suppressor effect, thereby accelerating ocular melanoma progression [17].

In addition to tumorigenesis, lactylation–RNA modification crosstalk plays a crucial role in sustaining tumor proliferation across multiple malignancies. Colony-stimulating factor 1 (CSF1) induces M2-like macrophage polarization by engaging its receptor CSF1R. In pancreatic ductal adenocarcinoma (PDAC), H3K18la stabilizes *CSF1* mRNA by activating *IGF2BP2* transcription, thereby promoting M2-like macrophage polarization and PDAC cell proliferation [65]. In CRC, METTL3-mediated m6A modification of *GLUT1* mRNA enhances its translational efficiency, leading to increased glucose uptake, lactate production, and mTORC1 signaling activation, thereby driving tumor growth [112]. In lung adenocarcinoma (LUAD), METTL3 catalyzes m6A modification of *ENO1* mRNA, while YTHDF1 recognizes the m6A-modified 359 A locus and facilitates ENO1 translation, resulting in increased glucose uptake, lactate and ATP synthesis, which fuels tumor cell proliferation and accelerates LUAD progression [100]. Similarly, in papillary thyroid carcinoma (PTC), the RNA-binding protein HNRNPC regulates PKM alternative splicing via m6A modification, shifting isoform expression toward PKM2 upregulation and PKM1 suppression. This metabolic switch reinforces the Warburg effect, thereby facilitating tumor cell proliferation [104].

Lactylation–RNA modification also promotes tumor cell migration, invasion, and metastasis. Pyruvate dehydrogenase kinase 4 (PDK4) facilitates glycolysis by phosphorylating and inhibiting the pyruvate dehydrogenase complex (PDC), thereby reducing pyruvate conversion to acetyl-coenzyme A (acetyl-CoA). The METTL16-IGF2BP1 complex indirectly upregulates PDK4 by stabilizing *SOGA1* mRNA, leading to increased glycolytic flux and lactate production, which in turn drives CRC invasion and migration [107]. In ocular melanoma, lactate-induced H3K18 lactylation activates *ALKBH3* transcription, promoting tumor cell migration and invasion. Mechanistically, ALKBH3 removes m1A modification from *SP100A* mRNA, a tumor suppressor, reducing its stability and translational efficiency. This disruption inhibits the formation of tumor-suppressive leukemia protein (PML) condensates, thereby weakening tumor suppression [70]. Similarly, in osteosarcoma, NAT10 mediates ac4C modification, enhancing the stability and translation of *YTHDC1* mRNA. YTHDC1 subsequently recognizes and binds to m6A-modified sites on *PFKM* and *LDHA* mRNAs, stabilizing these transcripts and promoting glucose uptake and lactate production. This metabolic adaptation fulfills the biosynthetic and energetic demands of osteosarcoma cells, fostering an invasive phenotype [98]. In bladder cancer, WTAP promotes m6A modification within the 3’ UTR of *PIGT* mRNA and interacts with IGF2BP2 to enhance transcript stability, thereby increasing PIGT expression. Consequently, PIGT regulates GLUT1 glycosylation and membrane trafficking, remodeling glucose transport to promote glycolysis and metastatic progression [115].

Tumor stemness refers to the ability of tumor cells to self-renew and differentiate into multiple lineages, driving tumorigenesis, drug resistance, recurrence and metastasis. Lactylation and RNA modification bidirectionally regulate tumor cell stemness. β-Catenin modulates cell proliferation, metastasis, and stemness by interacting with TCF/LEF transcription factors. In GC, H3K18la promotes *ATF5* mRNA degradation by increasing METTL14 expression, thereby inhibiting the ATF5-driven transcriptional activation of *WDR74* and preventing the nuclear translocation of β-catenin, ultimately suppressing tumor stemness [63]. In contrast, NSUN2 stabilizes *ENO1* mRNA via m5C modification, enhancing glycolysis and lactate production and thereby promoting tumor stemness in CRC [64].

Furthermore, lactylation and RNA modification also play dual roles in regulating cell death, including ferroptosis and cuproptosis, thereby shaping tumor progression. Tumor-derived lactate facilitates N-alpha-acetyltransferase 10 (NAA10)-mediated K508 lactylation of NSUN2, increasing its catalytic activity. NSUN2 lactylation enhances m5C methylation and stabilizes *GCLC* mRNA, thereby promoting glutathione (GSH) synthesis, reducing lipid peroxidation, and conferring ferroptosis resistance in tumor cells [76]. Moreover, ferredoxin 1 (FDX1) acts as a reductase that catalyzes the reduction of Cu^2+^ to the more toxic Cu^1+^, inducing cuproptosis. In GC cells, lactylation-modified METTL16 promotes cuproptosis by stabilizing *FDX1* mRNA and increasing FDX1 expression via m6A modification [73]. Conversely, NUDT21 lactylation promotes cuproptosis resistance by reprogramming APA to destabilize FDX1 mRNA [75].

### Reprogramme metabolism

Beyond glycolysis, recent studies suggest that lactate-derived lactylation and RNA modifications constitute a key regulatory axis that connects glycolysis with amino acid and lipid metabolism. Histone lactylation can upregulate YTHDC1 and drive lipid metabolic remodeling through NEAT1–SCD signaling [66], while lactylation of NSUN2 enhances glutathione synthesis via GCLC stabilization, linking epitranscriptomic regulation to amino acid metabolism [76]. In parallel, amino acid–derived glutamate can further amplify glycolysis through m6A-dependent HK2 expression [91]. Collectively, these findings highlight that lactylation- and RNA modification–mediated cross-talk orchestrates a broader metabolic reprogramming beyond glycolysis, encompassing both lipid and amino acid metabolic networks.

### Tumor immune escape

Tumor immune escape refers to the ability of malignant cells to evade immune surveillance and destruction and involves multiple pathways, such as impaired antigen delivery, immune checkpoint activation, and the establishment of the tumor immunosuppressive microenvironment. Tumor-infiltrating myeloid cells (TIMs) are myeloid cell populations that infiltrate the TME and contribute to tumor immune escape. Lactate induces H3K18 lactylation via p300, which activates *METTL3* transcription and upregulates its expression. METTL3 subsequently catalyzes the m6A modification of *JAK1* mRNA and enhances its translational efficiency through the m6A-YTHDF1 axis, leading to the activation of the JAK1/STAT3 signaling pathway. This pathway amplifies the immunosuppressive effects of TIMs and promotes the infiltration of tumor-associated macrophages (TAMs) and myeloid-derived suppressor cells (MDSCs), ultimately contributing to the formation of the immunosuppressive microenvironment [72]. In addition, ALKBH5 facilitates *MCT4* mRNA expression by removing its m6A modification, resulting in elevated lactate levels within the TME. The accumulation of lactate promotes the recruitment of regulatory T cells (Tregs) and MDSCs, thereby impairing antitumor immunity. Notably, ALKBH5 knockdown significantly reduces lactate accumulation and enhances tumor responsiveness to PD-1 blockade therapy [88]. In GC, IGF2BP3 recognizes m6A-modified *LDHA* mRNA, stabilizing its transcript and thereby promoting glycolysis and lactate production. Elevated lactate levels suppress CD8^+^ T-cell-mediated antitumor immunity, accelerating immune evasion in GC [108]. In addition to shaping an immunosuppressive microenvironment, lactylation and RNA modification drive immune escape by regulating immune checkpoint pathways. Specifically, the immune checkpoint molecule CD155 binds to (T cell immunoreceptor with Ig and ITIM domains) TIGIT, thereby suppressing T-cell and natural killer (NK) cell activity. Concurrently, METTL1 upregulates PKM2 expression and glycolysis; the nuclear translocation of the PKM2 dimer directly activates *CD155* transcription, reducing CD16^+^ NK cell infiltration and further facilitating immune escape in CRC [77].

### Drug resistance and adaptation

**Fig. 3:**
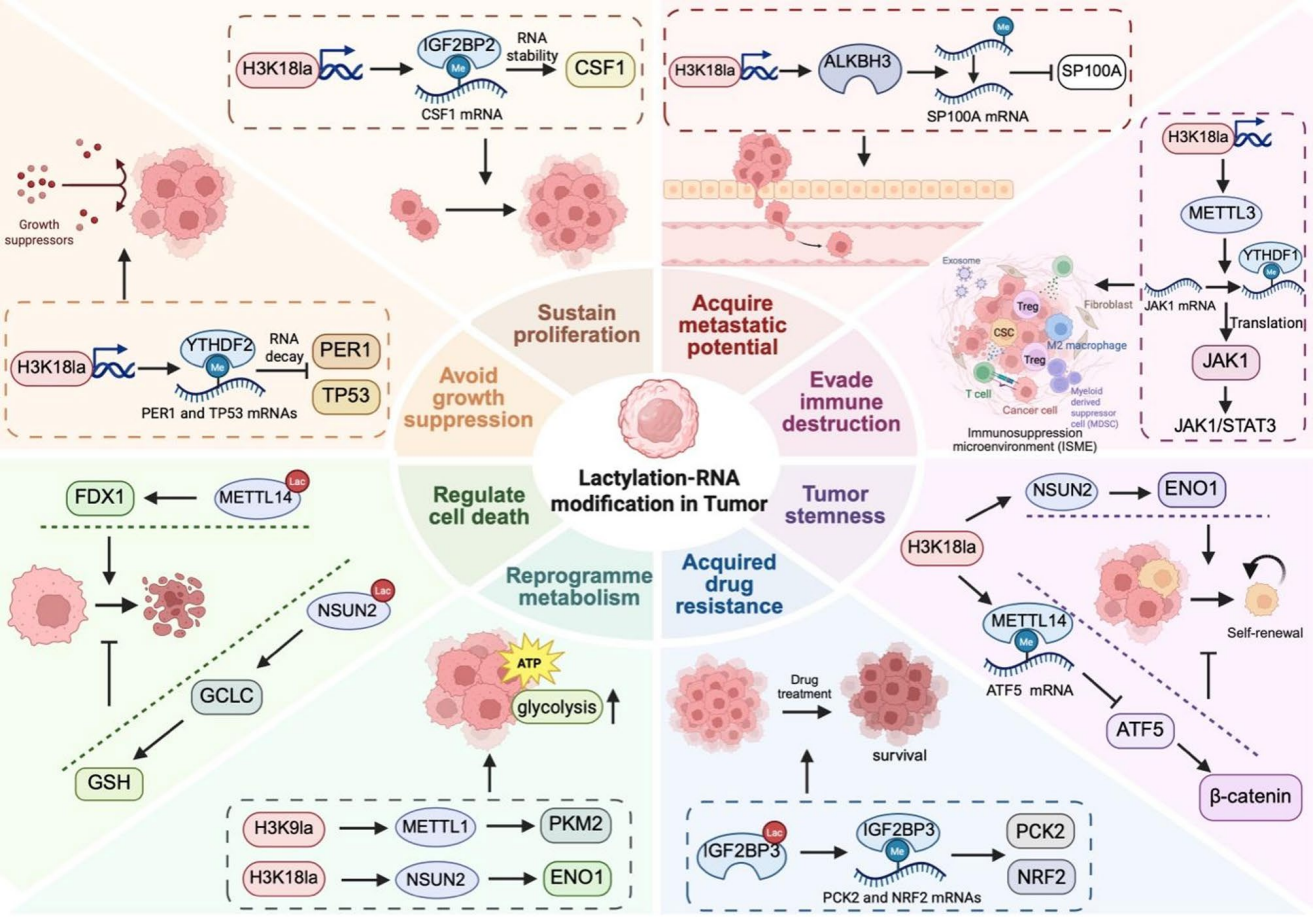
Lactylation-RNA modification crosstalk in tumor biology. Lactylation and RNA modifications coordinately regulate key hallmarks of cancer. Sustain proliferation: H3K18la enhances cell proliferation via the IGF2BP2-mediated stabilization of *CSF1* mRNA. Avoid growth suppression: H3K18la contributes to preventing growth suppression in melanoma cells by inducing the transcription of *YTHDF2*, which promotes the degradation of tumor suppressor transcripts such as *PER1* and *TP53*. Acquire metastatic potential: H3K18la upregulates ALKBH3, which reduces *SP100A* mRNA stability to facilitate metastasis. Evade immune destruction: H3K18la upregulates METTL3, which stabilizes *JAK1* mRNA and activates the JAK1–STAT3 pathway, promoting the infiltration of TAMs and MDSCs and suppressing antitumor immune responses. Tumor stemness: H3K18la suppresses β-catenin signaling and gastric cancer stemness through METTL14-induced degradation of *ATF5* mRNA, whereas in CRC, H3K18la promotes tumor stemness via NSUN2-mediated stabilization of *ENO1* mRNA. Acquired drug resistance: Lactylated IGF2BP3 enhances its binding to *PCK2* and *NRF2* mRNAs, promoting lenvatinib resistance in HCC. Reprogramme metabolism: H3K9la enhances aerobic glycolysis and lactate production via METTL1-mediated upregulation of PKM2, whereas H3K18la promotes NSUN2-mediated stabilization of *ENO1* mRNA. Regulate cell death: Lactylated NSUN2 stabilizes *GCLC* mRNA, thereby promoting glutathione (GSH) synthesis and conferring ferroptosis resistance. Conversely, in GC, lactylated METTL16 promotes cuproptosis by stabilizing *FDX1* mRNA. Created in BioRender. Liu, S. (2025) https://BioRender.com/h8krx8l

Tumor drug resistance remains a major obstacle in cancer therapy. Emerging evidence suggests that lactylation and RNA modification jointly contribute to resistance against chemotherapeutic and targeted agents. Mechanistically, such modifications have been implicated across multiple tumor types. Phosphoenolpyruvate carboxykinase 2 (PCK2), a key enzyme in gluconeogenesis, regulates redox homeostasis, whereas nuclear factor erythroid 2-related factor 2 (NRF2) elevates the cellular antioxidant stress response by activating antioxidant protein expression. In lenvatinib-resistant HCC cells, lactylation at K76 of IGF2BP3 upregulates the expression of PCK2 and NRF2 and restores redox homeostasis by increasing GSH and NADPH levels and reducing reactive oxygen species (ROS) production, thereby attenuating lenvatinib-induced apoptosis and promoting therapeutic resistance [74]. In ccRCC, METTL3 enhances glucose uptake and lactate accumulation by catalyzing the m6A modification of *GLUT1* mRNA, stabilizing its transcript and activating mTORC signaling, which drives resistance to mTOR inhibitors [114]. Additionally, METTL3 mediates m6A modification of the 5’ UTR of *PDK4* mRNA and promotes its translation and stabilization through interaction with the YTHDF1/eukaryotic elongation factor 2 (eEF-2) complex, driving glycolysis and ATP synthesis and leading to reduced susceptibility to doxorubicin (Dox) in both cervical and hepatocellular carcinoma [106] (Fig. 3).

## Targeted therapy

Lactylation and RNA modification are increasingly recognized as key epigenetic and metabolic regulators that drive tumor progression and have emerged as attractive therapeutic targets. Pharmacological disruption of the lactylation axis (e.g., LDHA inhibition) or modulation of RNA-modifying enzymes (e.g., methyltransferases and demethylases) can effectively reverse tumor-associated metabolic reprogramming and epigenetic dysregulation. Furthermore, combining these approaches with chemotherapy or immunotherapy has demonstrated synergistic potential. Nevertheless, single-pathway targeting strategies remain limited in clinical settings. The dual inhibition of lactylation and RNA modification by disrupting the metabolism-epigenetics regulatory network offers a promising avenue to overcome these constraints (Tables 3 and 4).

**Table 3:** Summary of targeted therapies and their current developmental status in cancer treatment

Cancer Type	Study model	Targeted Drug	Therapeutic Target Classification	Primary Target	Combination Strategy	Mechanism	Research Status	Refs.
**Colorectal Cancer**	MC38	STM2457 (METTL3 inhibitor)	RNA Modification Modulator	METTL3	Monotherapy (pre-treated BM-Mf and BM-MDSCs)	Epigenetic modulation	In vivo	[72]
	SW480, HT29; AOM/DSS mouse	Nsun2-i4 (NSUN2 inhibitor)	RNA Modification Modulator	NSUN2	Monotherapy or combination with anti-PD-1 therapy	Epigenetic modulation	In vitro and in vivo	[64]
	BALB/c; C57BL/6 mice; xenografts of HCT-116, RKO, and MC38	METTL1-WDR4-IN-1, Shikonin (PKM2 inhibitor)	Combination Therapy	METTL1, PKM2, CD155	Monotherapy or combination of METTL1-WDR4-IN-1 and Shikonin	Epigenetic & metabolic inhibition	In vivo	[77]
	HCT-116/5-FU, SW480/5-FU, SW620/5-FU; xenograft models	FX11 (LDHA inhibitor)	Metabolic Inhibitor	METTL3, LDHA	Monotherapy or combination of sh-METTL3 and FX11	Epigenetic & metabolic inhibition	In vitro and in vivo	[81]
	HCT116, DLD1; CRC organoids, HCT116 xenografts, Apc^Min/+^ mice	Sirolimus (Rapamune ^®^), Temsirolimus (Torisel^®^, mTORC1 inhibitors)	Combination Therapy	METTL3, mTORC1	Monotherapy or combination with sh-METTL3	Epigenetic & signaling inhibition	In vitro and in vivo	[112]
	HCT116, DLD1; nude mice xenografts	DAA (pan-methylation inhibitor)	RNA Modification Modulator	METTL3	Monotherapy	Epigenetic modulation	In vitro and in vivo	[86]
**Ocular Melanoma**	92.1, CRMM1	Oxamate, 2-DG	Metabolic Inhibitor	Histone lactylation	Monotherapy	Metabolic inhibition	In vitro	[70]
	OCM1, CRMM1, orthotopic xenograft mouse	2-DG, Oxamate, LDHA/B siRNAs	Metabolic Inhibitor	Histone lactylation	Monotherapy	Metabolic inhibition	In vitro and in vivo	[17]
**HCC**	Huh-7, HepG2	C646 (p300 inhibitor)	Lactylation Inhibitor	Histone lactylation, p300	Monotherapy	Epigenetic modulation	In vitro	[66]
	Post-treatment surgical HCC samples; orthotopic xenograft	2-DG, siIGF2BP3-loaded liposomes (si-LNPs)	Combination Therapy	IGF2BP3	Combination of lenvatinib with 2-DG or siIGF2BP3-LNPs	Epigenetic & Post-translational Regulation	In vivo and clinical sample analysis	[74]
	HepG2, HCC-PDX mouse model	Cpd-5 (ALDOA inhibitor)	Metabolic Inhibitor	ALDOA	Monotherapy	Metabolic inhibition.	In vitro and in vivo	[79]
**ESCC**	KYSE30 cell, KYSE30 xenograft	Stiripentol (Diacomit^®^, LDHA inhibitor), Elesclomol (copper ionophore)	Lactylation Inhibitor	LDHA, NUDT21, FDX1	Combination of Stiripentol with Elesclomol	Post-translational Regulation	In vitro and in vivo	[75]
**APL**	NB4-R1, NB4-R2; BALB/c nude mouse xenograft model	GRh2 (histone deacetylase inhibitor)	Lactylation Inhibitor	Histone lactylation	Monotherapy or combination with ATRA	Epigenetic modulation	In vitro and in vivo	[116]
**Gastric Cancer**	HGC-27; xenograft mouse model	Elesclomol (cuproptosis inducer), AGK2 (SIRT2 inhibitor)	Delactylation Inhibitor	METTL16	Combination of elesclomol and AGK2	Post-translational Regulation	In vitro and in vivo	[73]
**LUAD**	CDX models, PDX models, and KPE mouse model	DAA (pan-methylation inhibitor), 2-DG, ENOblock (ENO inhibitor)	Combination Therapy	m6A, ENO1	Monotherapy	Epigenetic & metabolic inhibition	In vivo	[100]
**Leukemia**	NOMO-1, U937	R-2HG	RNA Modification Modulator	FTO	Monotherapy	Metabolic inhibition	In vitro	[99]
**TNBC**	4T1 tumor-bearing mice	Remodelin (NAT10 inhibitor)	RNA Modification Modulator	NAT10	Monotherapy or combination with anti-CTLA-4	Epigenetic modulation	In vivo	[111]
**ICC**	HCCC-9810, HuCC-T1; HuCC-T1 allografts in nude mice	STM2457 (METTL3 inhibitor)	RNA Modification Modulator	METTL3	Monotherapy or combination with Gemcitabine	Epigenetic modulation	In vitro and in vivo	[113]
**Melanoma**	B16 cells; B16 mouse	ALK-04 (ALKBH5 inhibitor)	RNA Modification Modulator	ALKBH5	Monotherapy or combination with GVAX and anti-PD-1	Epigenetic modulation	In vitro and in vivo	[88]

### Targeting the lactate metabolism pathway

Targeting the glycolytic pathway can effectively curb tumor malignant progression and improve the efficacy of conventional therapies by reducing lactate production and subsequent lactylation. Specific strategies include targeting key glycolytic enzymes such as LDHA, HK, PFK and PKM2 [117]. Glycolysis inhibitors (e.g., 2-deoxy-D-glucose [2-DG], Oxamate) can interfere with glucose metabolism and lower lactate accumulation, thereby reversing tumor immune escape and mitigating therapy resistance [118, 119]. In GBM, for example, glycolysis inhibition decreases lactate-driven histone lactylation, downregulates the expression of immunosuppressive cytokines (e.g., IL-10), and restores CD8^+^ T-cell cytotoxicity, ultimately increasing responsiveness to immunotherapy [51]. Similar benefits have been observed in non-small cell lung cancer (NSCLC) [119]. LDHA inhibitors (e.g., Stiripentol) also exhibit sensitization potential. Stiripentol significantly enhances the chemosensitivity of GBM to temozolomide (TMZ) by reducing lactate-mediated H3K9 lactylation [120]. Stiripentol or LDHA knockdown also suppresses lactylation of the DNA repair factor NBS1, thereby impairing DNA damage repair and sensitizing tumors to cisplatin and radiotherapy [53]. In addition, Oxamate can enhance the antitumor activity of CAR-T cells by inhibiting CCR8 lactylation, highlighting the therapeutic potential of metabolism-immune axis co-targeting [121].

Despite these advances, tumors can circumvent single-target inhibition through metabolic compensation. For example, METTL3 and IGF2BP2 stabilize *HK2* and *GLUT1* mRNAs through m6A modification to maintain metabolic activity [86, 93], thereby undermining the efficacy of single-agent inhibitors. In addition, the small-molecule GLUT1 inhibitor STF-31 triggers off-target effects by simultaneously inhibiting NAMPT non-target proteins [122]. Long-term inhibition of glycolysis can disrupt physiological energy metabolism in erythrocytes and myocytes, causing side effects and affecting patient tolerance. Therefore, more precise multi-targeting strategies need to be developed to overcome metabolic plasticity in tumors and enhance therapeutic efficacy.

### Targeting lactylation modifications

Lactylation has emerged as a critical regulatory mechanism driving tumor progression and therapeutic resistance. This dynamic modification is catalyzed by lactyltransferases such as KAT8 and EP300, positioning these enzymes as compelling therapeutic targets. KAT8-catalyzed lactylation of eukaryotic elongation factor 1A2 (eEF1A2) at K408 enhances its GTPase activity, thereby boosting translation elongation and protein synthesis to support the rapid proliferation of colorectal cancer cells. The KAT8 inhibitor MG149 significantly reduces eEF1A2 lactylation and suppresses CRC growth [40]. Similarly, in CRC, lactate accumulation induces H3K18 lactylation, upregulates the autophagy-enhancing protein RUBCNL, and facilitates autophagosome maturation, enabling tumor cell survival under hypoxia and contributing to bevacizumab resistance. EP300 inhibition with A-485 effectively attenuates H3K18la, reverses autophagy-dependent resistance, and enhances the therapeutic efficacy of bevacizumab [123]. Collectively, these findings underscore the therapeutic potential of targeted lactylation.

Nevertheless, the donor-enzyme-substrate regulatory network underlying lactylation remains incompletely elucidated, and there is a substantial degree of overlap between lactylation- and acetylation-modifying enzymes [48], which may lead to off-target risks. For example, the inhibition of H3K18la in bladder cancer has been shown to restore cisplatin sensitivity [124]; however, owing to the shared catalytic machinery, such interventions may inadvertently disrupt H3K18 acetylation and interfere with essential transcriptional programs, leading to off-target effects. Therefore, future studies should focus on the development of highly selective inhibitors.

### Targeting RNA modifications

Accumulating evidence indicates that targeting core regulatory elements of RNA modifications can suppress tumor progression by modulating glycolytic metabolism. In CRC, METTL3 inhibition downregulates GLUT1 expression, impairs glycolysis and reduces lactate production, ultimately suppressing tumorigenesis [86, 112]. In addition, the targeting of RNA-modifying enzymes can attenuate therapeutic resistance by modulating the expression of glycolytic enzymes such as LDHA and PKM2. For example, in triple-negative breast cancer (TNBC), NAT10 enhances *JunB* mRNA stability through ac4C modification, drives the upregulation of LDHA expression, promotes glycolysis and lactate accumulation, inhibits T-cell activation and accelerates tumor progression, whereas the NAT10 inhibitor Remodelin reduces LDHA expression, remodels the tumor microenvironment, and effectively inhibits tumor growth and lung metastasis. Notably, combining Remodelin with CTLA-4 blockade further amplifies T-cell–mediated antitumor immunity by increasing CTLA-4 expression in T cells [111]. Similarly, ALKBH5 suppresses the JMJD8/PKM2 signaling axis by removing m6A modification of the *JMJD8* mRNA 3’ UTR, and reduces the activity of glycolysis and lactate production to inhibit the development of tumors. *ALKBH5* mRNA-loaded folic acid-modified exosome‒liposome hybrid nanoparticles restored its expression and significantly inhibited tumor progression in preclinical models [105].

However, RNA modification-targeting strategies face two principal challenges: RNA-modifying enzymes exhibit functional pleiotropy. For example, METTL3 modifies both mRNAs and tRNAs, leading to a lack of selectivity of existing inhibitors owing to redundant targeting. Second, RNA modifications exhibit dynamic spatiotemporal heterogeneity, with significant differences in enzyme expression, localization, targeting, and activity across tissue types [125]. This variability constrains the efficacy of single-target interventions.

### Dual-Targeted therapy

Crosstalk between lactylation and RNA modifications plays a pivotal role in driving tumor progression, immune evasion, and therapeutic resistance. The two modifications form a positive feedback loop by sharing regulatory nodes, and co-targeting can disrupt metabolic‒epigenetic crosstalk. Accordingly, simultaneous inhibition of lactylation and RNA modification represents a promising strategy for anticancer therapy. This can be achieved through the combination of lactylation or glycolysis inhibitors with RNA modification inhibitors, potentially increasing treatment sensitivity. For example, in 5-fluorouracil (5-FU)-resistant colorectal cancer, the METTL3 inhibitor STM2457 restores sensitivity to 5-FU by repressing glycolysis in CRC cells, whereas the LDHA inhibitor FX11 effectively reduces lactate production. Compared with sh-METTL3 or FX11 alone, combining FX11 with sh-METTL3 resulted in greater 5-FU sensitization [81]. Similarly, Preclinical data demonstrate that pharmacological inhibition of METTL1 using METTL1-WDR4-IN-1, in combination with the PKM2 inhibitor shikonin, significantly impairs tumor growth in murine CRC models, with greater efficacy observed than with either agent alone [77]​. These findings underscore the therapeutic potential of dual-targeted approaches involving RNA modifications and glycolysis/lactylation inhibition to disrupt metabolic‒epigenetic crosstalk and overcome therapy resistance.

**Fig. 4:**
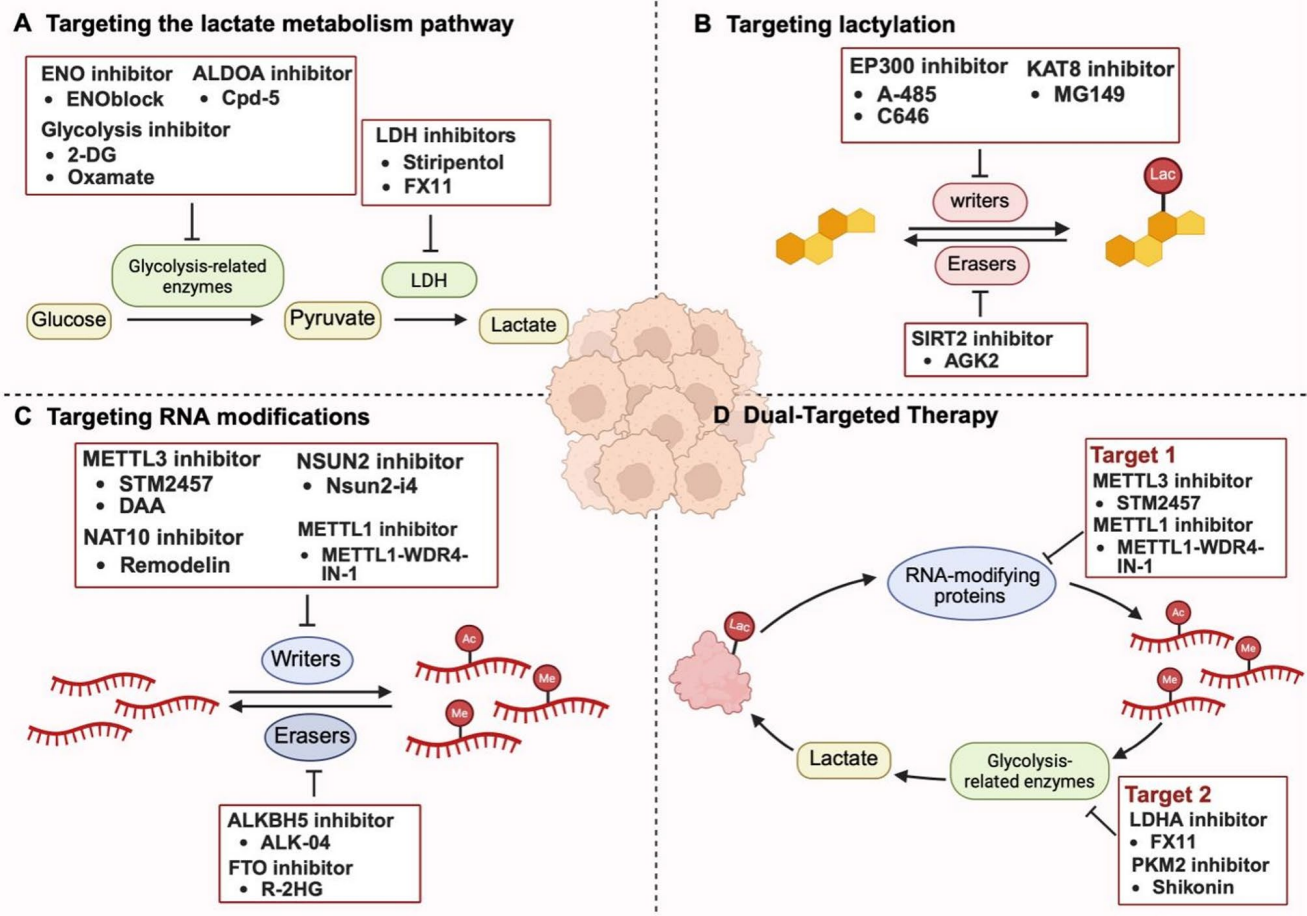
Therapeutic landscape targeting lactate metabolism, histone lactylation, and RNA modifications in cancer. **(A)** Strategies targeting lactate production include inhibitors of glycolysis-related enzymes such as ENO (e.g., ENOblock), ALDOA (e.g., Cpd-5), and glycolysis itself (e.g., 2-DG, oxamate), as well as LDH inhibitors (e.g., stiripentol, FX11), reducing the conversion of glucose to lactate that fuels tumor progression. **(B)** Lactylation is targeted by inhibiting lactylation writer enzymes, such as EP300 (e.g., A-485, C646) and KAT8 (e.g., MG149), and by inhibiting lactylation erasers like SIRT2 (e.g., AGK2), thereby regulating lactate-derived epigenetic and post-translational modification. **(C)** RNA modifications are modulated through inhibition of “writers” (e.g., METTL3 with STM2457 or DAA; NAT10 with remodelin; NSUN2 with Nsun2-i4; METTL1 with METTL1–WDR4–IN-1) and “erasers” (e.g., ALKBH5 with ALK-04; FTO with R-2HG), affecting RNA methylation (Me) and acetylation (Ac) levels. **(D)** A dual-targeted therapy simultaneously blocks lactate production (Target 2: LDHA inhibitor FX11, PKM2 inhibitor shikonin) and RNA modification (Target 1: METTL3 inhibitor STM2457, METTL1 inhibitor), disrupting the oncogenic crosstalk between lactylation and RNA modifications in cancer cells. Created in BioRender. Liu, S. (2025) https://BioRender.com/w6zh0ai

**Table 4:** Potential biomarkers associated with lactylation and RNA modifications in cancer

Cancer Type	Biomarker	Detection Method	Clinical Significance	Validation Status	Ref.
Hepatocellular Carcinoma	H3K9la, H3K56la	WB, IF	Promoting tumorigenesis	Clinical tissue and in vitro	[126]
	YTHDC1	IHC, WB	Promoting tumor progression	Clinical tissue, in vivo and in vitro	[66]
Gastric cancer	IGF2BP3	qRT-PCR, WB	Promoting tumor progression and immune evasion	Clinical tissue and in vitro	[108]
	AARS1	IHC, WB, qRT-PCR, IP, IF	Promoting tumor progression	Clinical tissue, in vivo and in vitro	[31]
Colorectal cancer	pan-Kla	WB, IF, IP	Promoting protein synthesis and tumorigenesis	Clinical tissue and in vitro	[40]
	Lactylation, H3K18la	IHC, WB, RNA-seq	Promoting CRC progression and bevacizumab resistance	Clinical tissue and in vitro	[123]
	METTL3	IHC, qRT-PCR, WB	Promoting tumor progression	Clinical tissue and in vitro	[72, 112]
	METTL16	IHC, qRT-PCR, WB	Promoting CRC progression	Clinical tissue, in vivo and in vitro	[95]
iCCA	METTL3	m6A dot blot, ELISA, RT-qPCR, WB, IHC	Promoting tumor progression	Clinical tissue and in vitro	[113]
	NCL hyper-lactylation	WB, IP, LC-MS/MS	Promoting ICC progression	Clinical tissue	[128]
NSCLC	pan-Kla, H3K18la	IHC, WB	Promoting immune escape	Clinical tissue and in vitro	[119]
Ocular melanoma	H3K18la	IF, WB, IP, IHC, MS	Promoting OM tumorigenesis	Clinical tissue and in vitro	[17]
Bladder cancer	ALKBH3	m1A dot-blot assay, WB, IHC, RNA-seq	Promoting tumor progression	Clinical tissue, in vitro and in vivo	[70]
Triple-negative breast cancer	NAT10	RNA-seq, qRT-PCR, IHC	Promoting TNBC progression and immunosuppression	Clinical tissue, in vitro and in vivo	[111]
Cervical cancer	METTL3	MeRIP-seq, RT-PCR	Promoting CC cell proliferation	Clinical tissue and in vitro	[78]
Osteosarcoma	NAT10	IHC	Promoting tumor progression	Clinical tissue	[98]

## Conclusions and perspectives

In recent years, the pivotal role of the crosstalk between lactylation and RNA modifications in tumor metabolic reprogramming and malignant phenotypic regulation has been gradually revealed. This review systematically summarizes the molecular mechanisms and biological functions of both lactylation and RNA modifications in cancer, and discusses their potential as therapeutic targets, either alone or in combination. The synergistic regulation of lactylation and RNA modification provides new perspectives for metabolic‒epigenetic studies and is highly important for understanding tumor development and refining targeted therapies.

However, several key scientific questions in this field remain unresolved. Although acetyltransferases and deacetylases have been implicated in catalyzing the reversible addition of lactyl-CoA to lysine residues, the enzymatic origin of lactyl-CoA itself remains unknown. In addition, how the “write-erase” dynamic balance of lactylation responds to metabolic stress within the TME and the functional heterogeneity of different lactylation sites need to be explored in depth.

Second, revealing the crosstalk between PTMs and nucleic acid modifications is essential for decoding the complex regulatory networks underlying tumor progression. Current studies suggest that lactylation and RNA modifications interact in multiple ways: lactylation modulates the expression and activity of RNA-modifying enzymes; RNA modifications regulate lactylation levels by influencing the glycolysis pathway; and a positive feedback loop reinforces their mutual regulation. However, most studies have focused on how RNA modifications affect glycolysis and lactate metabolism, while the mechanisms by which RNA modifications directly regulate lactylation remain poorly characterized and warrant further mechanistic dissection.

Therapeutically, targeting either lactylation or RNA modifications has demonstrated efficacy in inhibiting tumor progression and enhancing the effectiveness of chemotherapy and targeted therapies. Nevertheless, monotherapies are often limited by metabolic redundancy and compensatory pathways. The development of dual-targeted inhibitors with high specificity and minimal off-target effects is therefore urgently needed. Compared with single-agent approaches, combination strategies targeting both lactylation/glycolysis and RNA modifications may offer superior therapeutic benefits, representing a promising direction for cancer therapy.

Finally, advances in high-throughput technologies have enabled increasingly precise detection of both RNA modifications and lysine lactylation. For RNA modifications, immunoprecipitation-based sequencing methods such as MeRIP-seq, acRIP-seq, and m6A-seq are widely used, yet often suffer from limited resolution and antibody specificity [129]. Recent progress in direct RNA sequencing and enzyme-assisted detection strategies offers improvements in quantification and site resolution [130]. For lactylation, detection primarily relies on LC-MS/MS analysis and pan-Kla antibody enrichment, but these approaches face challenges such as low stoichiometry, poor antibody specificity, and ambiguous site localization [131]. A notable advance is the use of diagnostic ions in MS/MS, which allows antibody-free detection of lactylated peptides, increasing confidence and proteome coverage [33]. In addition, single-cell and spatial transcriptomic platforms are emerging as powerful tools to study the heterogeneity and spatial context of RNA modifications and lactylation [132], For example, studies integrating scRNA-seq with spatial analysis in hepatocellular carcinoma have mapped histone lactylation-associated gene expression at single-cell resolution, revealing cell-type-specific patterns and prognostic implications [133]. Further development of high-resolution and multiplexed approaches will be essential to overcome these challenges.

## Data Availability

No datasets were generated or analysed during the current study.

## Abbreviations

AARS1: Alanyl-tRNA synthetase 1
ac4C: N4-acetylcytosine
AML: Acute myeloid leukemia
ALI: Acute lung injury
APA: Alternative Polyadenylation
APL: Acute promyelocytic leukemia
As-IPF: Arsenite-related idiopathic pulmonary fibrosis
ATAT1: α-tubulin acetyltransferase 1
ALDOA: Fructose-bisphosphate aldolase A
BUB1B: BUB1 mitotic checkpoint serine/threonine kinase B
CRC: Colorectal cancer
CC: Cervical cancer
ccRCC: Clear cell renal carcinoma
c-Myc: Cellular myelocytomatosis oncogene
CSF1: Colony-stimulating factor 1
DAA: 3-Deazaadenosine
DR: Diabetic retinopathy
ENO1: Enolase 1
ESCC: Esophageal squamous cell carcinoma
FDX1: Ferredoxin 1
GC: Gastric cancer
G6PT: Glucose-6-phosphate translocase
GLUT: Glucose transporter
GLUT1: Glucose transporter 1
GAPDH: Glyceraldehyde-3-phosphate dehydrogenase
GBM: Glioblastoma
GSH: Glutathione
GPI: Glucose-6-phosphate isomerase
H3K18la: H3K18 lactylation
H3K9la: H3K9 lactylation
HCC: Hepatocellular carcinoma
HK: Hexokinase
HK2: Hexokinase 2
HIF-1α: Hypoxia-inducible factor 1α
HSCs: Hepatic stellate cells
iCCA: Intrahepatic cholangiocarcinoma
IF: Immunofluorescence
IFN-β: Interferon β
IHC: Immunohistochemistry
IP: Immunoprecipitation
ISME: Immunosuppressive microenvironment
KL-la: Histone lysine L-lactylation
KSHV: Kaposi’s sarcoma-associated herpesvirus
LC-MS: Liquid Chromatography–Mass Spectrometry
LDH: Lactate dehydrogenase
LDHA: Lactate dehydrogenase A
LUAD: Lung adenocarcinoma
LGSH/SLG: S-D-lactoylglutathione
Lactyl-CoA: Lactyl-coenzyme A
LLPS: Liquid‒liquid phase separation
LND: Lonidamine
MCT: Monocarboxylate transporter
MCT4: Monocarboxylate transporter 4
m6A: N6-methyladenosine
m5C: 5-methylcytidine
m1A: N1-methyladenosine
m7G: N7-methylguanosine
MM: Multiple myeloma
MDSCs: Myeloid-derived suppressor cells
MS: Mass spectrometry
NAA10: N-α-acetyltransferase 10
NCL: Nucleolin
NSCLC: Non-Small Cell Lung Cancer
OC: Ovarian Cancer
OM: Ocular melanomas
OS: Osteosarcoma
PTM: Post-translational modification
PDAC: Pancreatic ductal adenocarcinoma
PDX: Patient-derived xenograft
PFK-1: Phosphofructokinase-1
F1,6BP: Fructose-6-phosphate to fructose-1,6-bisphosphate
PFKM: Phosphofructokinase muscle type
PK: Pyruvate kinase
PDK4: Pyruvate dehydrogenase kinase 4
PEP: Phosphoenolpyruvate
PKM2: Pyruvate kinase M2
PTC: Papillary thyroid carcinoma
PGK1: Phosphoglycerate kinase 1
qRT-PCR: Quantitative Reverse Transcription Polymerase Chain Reaction
R-2HG: R-2-hydroxyglutarate
TTK: TTK protein kinase
TIMs: Tumor-infiltrating myeloid cells
TNBC: Triple-negative breast cancer
Tregs: Regulatory T cells
TME: Tumor microenvironment
WB: Western blot
2-PGA: 2-phosphoglycerate
2-DG: 2-deoxyglucose
5-FU: 5-fluorouracil
